# Luminance contrast provides metric depth information

**DOI:** 10.1098/rsos.220567

**Published:** 2023-02-15

**Authors:** Paul B. Hibbard, Ross Goutcher, Rebecca L. Hornsey, David W. Hunter, Peter Scarfe

**Affiliations:** ^1^ Department of Psychology, University of Essex, Colchester, Essex, UK; ^2^ Psychology Division, Faculty of Natural Sciences, University of Stirling, Stirling, UK; ^3^ Department of Computer Science, Aberystwyth University, Aberystwyth, UK; ^4^ School of Psychology and Clinical Language Sciences, University of Reading, Reading, Berkshire, UK

**Keywords:** vision, stereopsis, image statistics, binocular disparity, shape from shading

## Abstract

The perception of depth from retinal images depends on information from multiple visual cues. One potential depth cue is the statistical relationship between luminance and distance; darker points in a local region of an image tend to be farther away than brighter points. We establish that this statistical relationship acts as a quantitative cue to depth. We show that luminance variations affect depth in naturalistic scenes containing multiple cues to depth. This occurred when the correlation between variations of luminance and depth was manipulated within an object, but not between objects. This is consistent with the local nature of the statistical relationship in natural scenes. We also showed that perceived depth increases as contrast is increased, but only when the depth signalled by luminance and binocular disparity are consistent. Our results show that the negative correlation between luminance and distance, as found under diffuse lighting, provides a depth cue that is combined with depth from binocular disparity, in a way that is consistent with the simultaneous estimation of surface depth and reflectance variations. Adopting more complex lighting models such as ambient occlusion in computer rendering will thus contribute to the accuracy as well as the aesthetic appearance of three-dimensional graphics.

## 1. Introduction

We inhabit a three-dimensional world, and our visual experience of this is created from the two-dimensional images that are projected into our two eyes. This experience is possible through the use of multiple visual cues to depth [1]. One important class of these depends on projective geometry—the way that three-dimensional structures project into two-dimensional images. This gives rise to many of the most familiar depth cues such as binocular disparity, motion parallax, linear perspective and image texture. Projective cues depend on simple geometric principles. Their role in visual perception is therefore relatively well-understood, and their study has tended to dominate research into the perception of depth and distance [2].

A second important set of depth cues consists of those that depend on the lighting of a scene and the reflectance of surfaces. This leads to the impression of depth from light and shade or *chiaroscuro*. While these cues have been exploited by artists since at least Renaissance times, their exact role in the perception of depth is much less clear [2]. This is because they depend on the way that light is reflected by the fine-grained structure of surfaces, and from one surface to another. They are thus complex to model, and their precise simulation depends on modern, high-powered graphics-rendering hardware and software. As a result, our understanding of these cues in vision science to date is less well established than that of projective cues [2].

**Figure 1.**
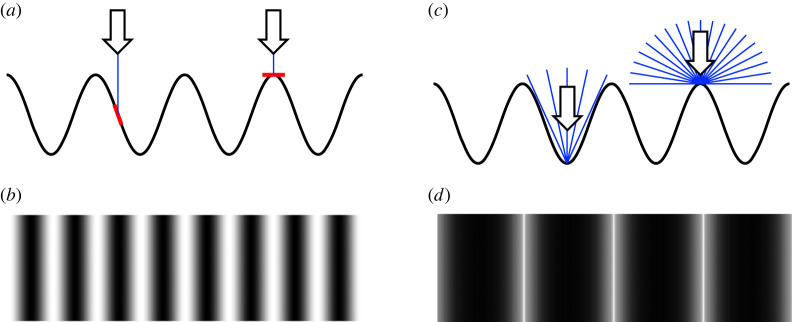
(*a*) In traditional models of shape-from-shading, under the assumption of a directional light source, luminance depends on the orientation of the surface relative to the direction of the light source. (*b*) The resulting image has its brightest points where the surface is normal to the lighting direction and its darkest points where the surface normal is rotated furthest away from the lighting direction. In this image, pixel luminance is proportional to the cosine of the angle between the surface and the light source. (*c*) Under the assumption of diffuse lighting, luminance depends on the area of the light source to which a point is exposed. Light from some directions will be occluded from points that are in crevices in the surface, as shown on the left. (*d*) These more distant, occluded points will appear darker in the image, while the points that are locally the closest to the observer will appear brighter. This image was created using a Monte Carlo ambient occlusion model, in which pixel luminance is proportional to the area of the diffuse light source to which it is exposed.

Variations in luminance do nevertheless provide important contributions to the perception of depth. This is evident for example in the perception of shape from shading [3]. Under the assumptions that light comes from a single direction [4,5], and that the observer is viewing a matte surface, the luminance of a point on a surface is directly related to its orientation relative to the light source [3] (figure 1*a,b*).

By contrast, under diffuse lighting of equal intensity from all directions, the luminance of a point depends on the area of the light source, such as the sky, to which it is exposed (figure 1*c,d*) [6]. As a result, points within crevices and concavities, which are partially shielded from the light source, will tend to be darker. As can be seen in figure 1, and as demonstrated by Tyler [7], these differences in lighting conditions will create luminance profiles with a very different shape, and a different spatial periodicity, for a sinusoidal depth profile. Since these points will also be more distant than other points in the local neighbourhood, this creates a local negative correlation between the distance and luminance of points. This ‘dark-is-deep’ relationship [7] can be used to identify convexities and concavities ('hills' and ‘valleys') on a surface. Leonardo da Vinci noted that artists employ this dark-is-deep strategy to convey a sense of three-dimensional surface structure [8,9]. In computer graphics, this technique is known as ambient occlusion. In comparison with more traditional techniques, based on the assumption of a collimated light source from a single direction, it is known to increase the realism and depth appearance of scenes [10–13]. Different assumptions underlie depth from luminance variations under directional and diffuse lighting, and both may be present in a single scene. They also provide independent cues to three-dimensional shape, the former being used to estimate surface orientation and the latter relative depth differences [14]. Although the relationship between luminance and distance under diffuse lighting is unreliable [15], it is evident in natural images. By analysing luminance and range data captured using a laser range finder, a negative correlation between relative distance and luminance was found within local areas of the scene [16].

In natural viewing, both directional and diffuse light sources may both be present. This means that the perception of shape from shading should ideally incorporate both components, which can be used to estimate the local orientation of surfaces, and global illumination considerations such as the dark-is-deep heuristic, in which local variations in luminance can be used to estimate relative depth.

Consistent with this, luminance variations have been shown to contribute to apparent depth in the manner predicted by the dark-is-deep correlation [17]. In this experiment, dark regions of a sinusoidal luminance profile were judged as farther away than bright regions. The stimuli also contained variations in binocular disparity, at the same frequency as the luminance variations, such that the brighter regions were assigned near disparities. Contrast and disparity were manipulated independently so that in most stimuli they signalled inconsistent depth variations. The modulation of binocular disparity did not contribute to perceived depth. These results are seemingly inconsistent with the mandatory combination of depth from luminance contrast with depth from binocular disparity according to a standard, maximum-likelihood cue-combination rule [18]. However, binocular disparity provided a very weak cue in this study, because the only luminance variation was the low-frequency sinusoidal modulation. This means that perceived depth would have been dominated by luminance. In a different study, both directional and diffuse lighting were present, and binocular information was made more reliable by the inclusion of a surface texture that provided more precise disparity cues [19]. In this case, the two cues were combined optimally. These results showed that the combination of binocular disparity and shading cues is consistent with the standard cue-combination model, but did not isolate the influence of the luminance-depth correlation on the perception of depth. The sign of the correlation between luminance and depth also influenced sensitivity in simple categorical judgements of which of two targets in a cluttered scene was closer [20].

The expected negative correlation can be exaggerated or diminished by manipulating the luminance of pixels in proportion to their distance from the observer [21]. In this study observers were asked to judge which of these manipulated images gave a better sense of the three-dimensional scene. Increasing the negative correlation between luminance and distance increased the sense of viewing a three-dimensional scene while reducing the correlation reduced it. This experiment did not directly measure the amount of depth perceived. This is an important distinction since stimulus manipulations that affect the apparent depth realism of a stimulus do not always increase the amount of depth perceived [22–24]. Of most relevance to the interaction of shape from shading with other depth cues, manipulations of shading that increase the sense that the observer is looking at a real three-dimensional surface can also reduce the amount of depth variation seen in the surface [24]. Depth realism is a qualitative judgement, and there may be multiple factors that may influence participants' responses [25]. For example, variations in this qualitative judgement have been proposed to be related to the precision with which depth judgements can be made [26], the degree of conflict between cues [27], whether information is scaled to provide absolute rather than relative depth [22], and to the perception of material properties such as shininess, glossiness and roughness [22]. Experiments to distinguish between these factors require comparisons targeted at the specific hypotheses and precisely worded instructions. In the current study, however, the focus is only on understanding how disparity and shading cues contribute to the magnitude of perceived depth.

These studies show that three-dimensional perception is influenced by the correlation between luminance and depth, but do not establish it as a quantitative depth cue that is combined with other cues. Some previous evidence [16] comes from stimuli with very imprecise binocular disparity cues, thus reducing the opportunity for them to contribute to depth [18,28,29]. Other studies have not measured depth perception directly, but rather the quality of three-dimensional experience [21]. The combination of luminance contrast and disparity cues in the perception of depth, and quantification of the relative contributions of the two cues, has been performed [30]. The stimuli in this study were randomly spaced vertical bars that sampled a Gaussian depth profile defined by variations in luminance, disparity or both, and the participant's task was to judge the horizontal position of the peak of the profile. Localization accuracy was much more precise when depth was defined by disparity. When both disparity and luminance cues were available, participants were unable to localize the peak of the profile when disparity and luminance had opposite signs of depth, and the magnitude of disparity was such that it nulled the luminance-defined depth. In this case, the surface appeared flat. This result demonstrates the combination of disparity and luminance cues into a single depth estimate and quantifies the relationship between depth specified by the two cues for the stimuli that were used.

In the current study, we directly assess the contribution of the negative luminance–distance correlation as a depth cue by artificially manipulating this relationship while keeping other properties of the image constant. If this correlation is used in the same way as other cues such as binocular disparity, it is necessary to establish both that luminance contrast contributes to the magnitude of perceived depth, and that it is combined with other depth cues. One way in which depth from shading could be combined with depth from other cues is through a weighted averaging, with the weights of the cues determined by their relative reliabilities [18]. For example, if binocular disparity (*B*), luminance contrast (*C*) and other cues (*O*) are averaged to estimate apparent depth, then depth settings (*D*) would be determined by:

1.1 $$D=|w_{d}s_{b}B+w_{c}s_{c}C+w_{O}O|,$$

where *w*_d_, *w*_c_ and *w*_o_ specify the weights for disparity, contrast and other cues (and $w_{d}+w_{c}+w_{o}=1$) and *s*_B_ and *s*_C_ determine the scaling between depth, and binocular disparity and contrast, respectively. These scaling factors are the necessary cue promotion step in the standard cue combination model, that is used to transform the available information (measured in degrees of retinal disparity, and unitless values of Michelson contrast) into a common measure of depth (measured in mm) [18]. ‘Other cues’ here is used to accommodate the broad range of other depth cues that may be available in any given scene. This model of combining depth cues assumes that estimates from individual cues are unbiased, with Gaussian uncertainty. When the information in a stimulus is consistent across cues, this will lead to an increase in precision, but will not affect the magnitude of depth perceived. When a conflict between cues is artificially introduced, the predicted outcome is an increase in precision, and a depth estimate that falls with the range of estimates from individual cues. Other models of cue combination make different predictions as the number of available cues is increased. For example, Tyler's accelerated cue combination principle predicts that perceived depth will increase with the number of depth cues available, asymptoting to veridical perception under full cue conditions [31]. This model also however predicts that the overall estimate of depth will tend to increase if the depth specified by the individual cues is increased.

We refer to the standard cue-combination model, in which the two cues are averaged regardless of their consistency, as mandatory combination. When the directions of depth signalled by disparity and contrast are the same, it predicts that increasing contrast while keeping disparity fixed will increase perceived depth. Conversely, if the two cues signal opposite directions of depth, it predicts that increasing contrast while keeping disparity constant will decrease perceived depth.

By contrast to depth cues such as binocular disparity, which signals variations in depth unambiguously, the dark-is-deep correlation may best be viewed as a heuristic rather than an unambiguous cue to depth. While the assumptions of diffuse lighting mean that locally more distant points will tend to be darker, variations in luminance are also caused by variations in the reflectance of the surface. When luminance variations are not consistent with other depth cues, this may be taken to signal that they represent variations in reflectance, rather than depth. In such cases luminance and colour variations should not contribute to the perception of depth [32–34]. We thus predict that the local luminance variations within an object will influence the perception of depth, but that global differences in luminance between objects will not.

Causal inference and other models of cue combination have been developed to infer whether cues originate from the same source, and should thus be combined [31,35]; alternatively, this decision can result directly from the cue combination process itself [36–38]. In the current case, the disambiguation that needs to be made is to determine whether luminance variations in the image result from depth or reflectance variations on the surface. Here, we consider a case where only binocular disparity and shading cues are available. We assume that the luminance variations will be interpreted as a cue to depth (when they are consistent with disparity) or reflectance (when they are in conflict with disparity), but not both. We refer to this model as consistency-dependent combination. In this case, estimated depth is determined by:

1.2 $$D=\{\begin{array}{c} \left|w_{d}s_{B}B+(1-w_{d})s_{C}C,|,\;\;\mathrm{if}\;\mathrm{sgn}(B)=\mathrm{sgn}(C\right)\\ |w_{d}s_{B}B|,\;\;\mathrm{otherwise}. \end{array}$$

**Figure 2.**
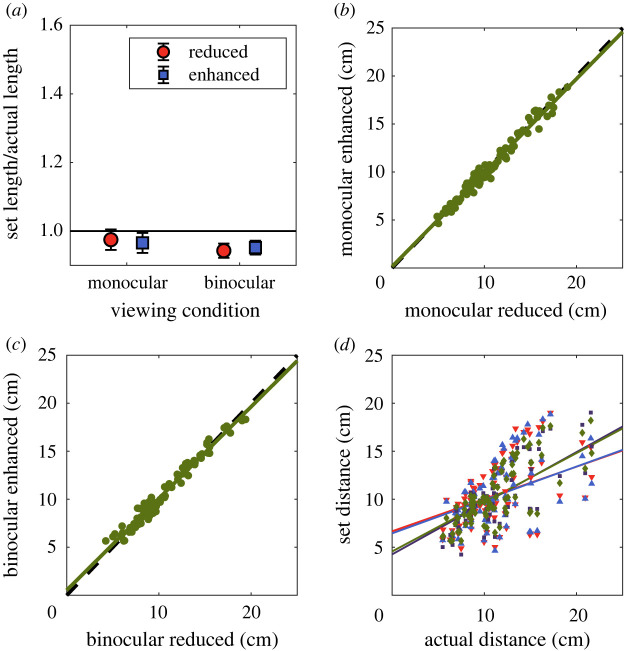
(*a*) Set distance ratios for the global, between object condition. Observers tended to underestimate distance, but there was no effect of luminance manipulation or the presence of binocular depth cues. Scatterplots of the settings in the enhanced versus reduced condition are shown in (*b*) for monocular viewing and (*c*) for binocular viewing. (*d*) Scatterplot of distance settings in all conditions against the ground truth (legend: q monocular reduced, p monocular enhanced, ¢ binocular reduced, u binocular enhanced). Error bars represent ±1 standard deviation.

When disparity and luminance depth cues are consistent, this model predicts that estimated depth increases as both disparity and contrast increase, following the standard cue-combination model. However, when the two cues are not consistent, it predicts that estimated depth will increase with disparity, but be unaffected by contrast (figure 2). The weight for the disparity cues was kept at *w*_d_ rather than 1 in the inconsistent condition. This reflects a belief that shape from shading is still available as a depth cue, but indicates a uniform surface. Alternatively, if the cue was considered to be absent, its weight would be set to zero, the binocular weight set to 1, and perceived depth would be predicted to increase. The form of this equation is very similar to Tyler's ‘Gregorian attractor’ [39], as a way of instantiating perceptual hypothesis testing [40]. In this case, the hypothesis under scrutiny is whether luminance variations result from the depth structure of the surface, or to spatial variation in reflectance.

We tested the role of the dark-is-deep heuristic as a metric depth cue, and how it is combined with other cues, in two ways. In our first experiment, we used stimuli that were created by capturing three-dimensional scans of natural objects, then arranging these into cluttered scenes. These stimuli, in which we know the luminance and three-dimensional location of each pixel, and the object in the scene to which it belonged, allowed us to manipulate and measure the influence of local luminance variations on perceived depth while keeping all other cues constant [21].

**Figure 3.**
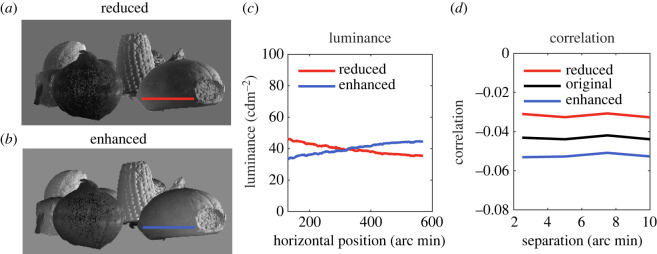
In the within-object condition, the luminance of individual pixels was made brighter or darker, depending on its distance relative to other points on the same object. (*a*) In the reduced condition, closer points were made darker and farther points brighter. (*b*) The opposite manipulation was made in the enhanced condition. (*c*) A horizontal sample through the images (shown by the lines in (a) and (b)) indicates luminance reducing as the surface gets closer to the observer in the reduced condition, and increasing in the enhanced condition. (*d*) Our original scene contained a negative correlation between the depth difference and luminance difference between pairs of pixels from the same object. The size of this correlation was increased in our enhanced condition, and decreased in our reduced condition.

In the first, within-object condition, the luminance of all points within each object was altered, dependent on its distance from the observer when compared with all other points within that object (figure 3). This allowed us to enhance the within-object, negative correlation between luminance and distance, by making nearer points lighter and farther points darker. On other trials, this correlation was reduced.

**Figure 4.**
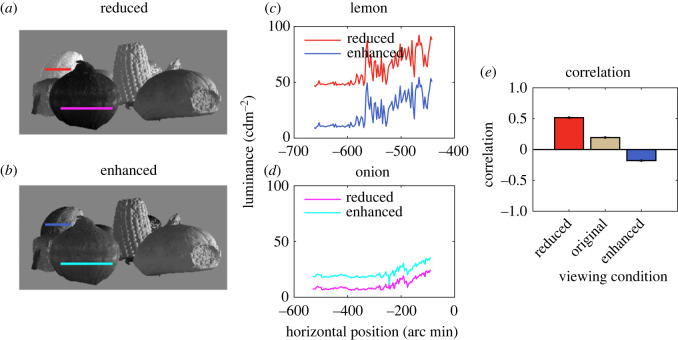
In the between-object condition, the manipulation of luminance was based on the distances of all points in an object considered together, to create (*a*) reduced and (*b*) enhanced conditions. (*c*) shows a luminance sample in the reduced and enhanced condition for a far object (the lemon) and (*d*) shows samples for a close object (the onion). In the reduced condition, the far object is made brighter and the close object darker in comparison with the enhanced condition. (*e*)There was a positive correlation between the mean luminance and distance of objects. This positive correlation increased in our reduced condition, and was negative in our enhanced condition.

In the second, between-object condition, the luminance of each point was increased or decreased depending on whether the object was relatively near or far in the scene (figure 4). We did not predict an effect on perceived depth in this case, however, for two reasons. The first is that the dark-is-deep correlation depends on the *local* effects of three-dimensional structure on variations in luminance contrast [16]. The second is that, while the reflectance properties of a surface are relatively constant within an object, there will be large variations in reflectance between objects. We thus predicted that the manipulation of the overall lightness of entire objects was more likely to be interpreted as a change in reflectance, and not to affect perceived depth [41].

**Figure 5.**
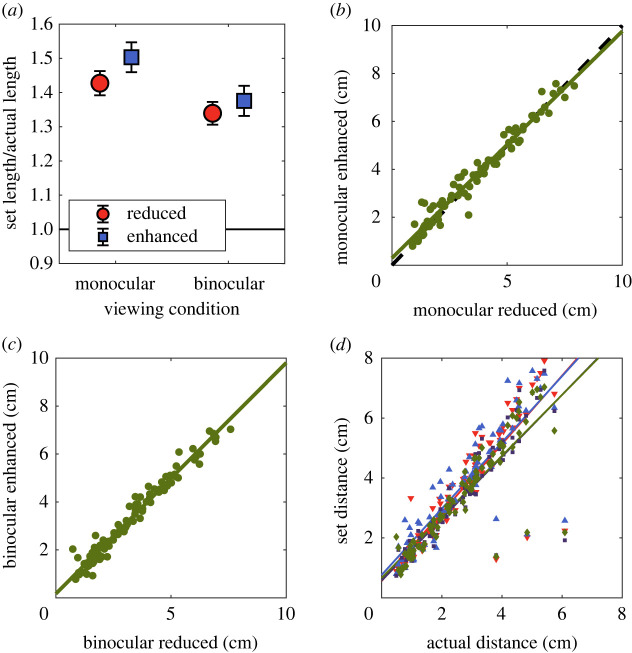
(*a*) Mean ratio of set distance to actual three-dimensional distance for the local, within-object condition. Settings were larger in the enhanced condition with monocular depth cues, but not with binocular depth cues. Settings were smaller, and more accurate when binocular depth cues are available. Scatterplots of the settings in the enhanced versus reduced condition are shown in (*b*) for monocular viewing and (*c*) for binocular viewing. (*d*) Scatterplot of distance settings in all conditions against the ground truth (legend: q monocular reduced, *p* monocular enhanced, ¢ binocular reduced, u binocular enhanced). Although in this plot the data cluster around the identity line, there is a significant change that is difficult to discern given the range of distance settings and the generally good performance on the task. This plot is included to demonstrate the additive nature of the effects of changing the luminance/depth relationship, and the effect of the other depth cues contained in the stimuli. Error bars represent ±1 standard deviation.

In our second experiment, we used random dot stereograms in which both the binocular disparity and the luminance of individual dots varied, in order to precisely manipulate the contribution of luminance as a cue to depth (figure 5). We varied the sign of the two cues so that they provided consistent or inconsistent depth information. We predicted that perceived depth would increase with increasing disparity in all conditions. By contrast, we predicted that perceived depth would increase with luminance contrast when its depth interpretation was consistent with disparity, but have no effect when it was in conflict.

## 2. Experiment one

The first experiment measured the contribution of luminance statistics to the perception of depth in scenes containing pictorial and binocular depth cues. To do this, we created scenes with known three-dimensional structure, and enhanced or reduced the correlation between depth and luminance. Objects were three-dimensional mesh models, obtained using a laser scanner. This allowed for ground truth knowledge of the three-dimensional structure for all stimuli (see methods for further details). Correlation between depth and luminance was enhanced or reduced by artificially lightening or darkening individual pixels, depending on whether they were relatively close to or farther from the observer [21].

In the first, within-object condition (figure 3), luminance was manipulated separately for pixels belonging to individual objects. Our scenes exhibited the expected local negative correlation between luminance and depth, which increased as the separation between the points increased (figure 3*c*). This correlation decreased in our ‘reduced’ condition and increased in our ‘enhanced’ condition. We expect luminance statistics to act locally as a cue to depth [16], and thus predict more depth to be perceived with an enhanced correlation, and less depth with a reduced correlation.

In the second, between-object condition (figure 4), the luminance of pixels was varied dependent on whether the objects to which they belonged were relatively close or far away in the scene. This lightened or darkened all pixels within a given object in the same way. In our original scenes, there was a positive correlation between luminance and distance (figure 4*e*). This illustrates that the luminance–depth relationship is not reliable as a global distance cue, depending as it does on surface reflectance variations between objects. This positive correlation increased in our ‘reduced’ condition, and became negative in our ‘enhanced’ condition, indicating that our manipulation had the desired effect.

Since the luminance of individual pixels in an image will depend on the reflectance of the object (the surface material) as well as the lighting conditions, our manipulation is consistent with an overall change in the reflectance of the object. We predict that our global luminance manipulation will not affect perceived distance.

### 2.1. Methods

#### 2.1.1. Materials

Stimuli were presented on a ViewPixx three-dimensional monitor, viewed from a distance of 50 cm. One pixel subtended 2.2 arc min. The luminance response was linearized using a Minolta LS-110 photometer; the luminance range of the display was 100 cd m^−2^. Observers' responses were captured using the computer keyboard and mouse. Stimuli were created using MATLAB and the Psychophysics Toolbox [42–44] using Windows 7.

Objects were scanned using a NextEngine three-dimensional Scanner and Multidrive turntable. Scanning settings were a 360° rotation with eight divisions, two tilt settings of ± 20 degrees, 62 points mm^−2^ resolution and the macro distance range. Once scanned, each object was edited in NextEngine Scan Studio HD. Meshes were fused with a 0.064 mm tolerance to create a water-tight model. Files were then saved as a .ply file, and converted into Alias Wavefront Object (.obj) files via MeshLab. Stereoscopic images were rendered using OpenGL via MATLAB using the Psychophysics Toolbox and presented against a grey background. OpenGL lighting with a (0.3 0.3 0.3) magnitude ambient component and a (0.7 0.7 0.7) diffuse component was used. In OpenGL, the ambient component does not depend on the depth structure of the scene, and does not, therefore, provide the approximation to global illumination implemented in ambient occlusion. The diffuse component provides the contribution that depends on the orientation of the surface relative to the directional light source, which in this case was a spotlight located 50 cm to the right of the scene, and directed at the centre of the screen. The purpose of the current experiment was not to test the effects of changes to the lighting model or illuminant used but to directly manipulate the correlation between depth and luminance. Objects were positioned at random locations within a 30 × 30 cm region of a horizontal plane, at a distance of 50 cm directly in front of the observer, subject to the constraint that there was no overlap in the three-dimensional bounding box with other objects in the scene. Binocular image pairs were created by rendering the scenes from two locations separated by an intercamera distance of 64 mm, close to the human average interpupillary distance of 63 mm [45,46], and with the projection screen distance aligned with the display screen distance of 50 cm. This created stereoscopic pairs in which the disparities accurately specified the three-dimensional structure of the scene.

Each pixel in the scene was labelled as belonging to either the background or to one of the objects. In the within-object condition, for each object, the luminance of each pixel *i* was manipulated according to the following equation:

2.1 $$L_{i}=L_{O}\left(1\pm \alpha \frac{Z_{i}-Z_{\mathrm{median}}}{Z_{\max}-Z_{\min}}\right),$$

where *Z*_median_, *Z*_max_ and *Z*_min_ are the median, maximum and minimum distances for that object, *L*_0_ is the luminance of each pixel before manipulation, and *α* is a gain parameter determining the degree of manipulation. A value of *α* = 0.1 was used, and positive distance values indicated points that are further from the observer. Relative to the median distance, luminance was subtracted from far points and added to near points in the enhanced condition, and the opposite for the reduced condition. Here, luminance is measured in cdm^−2^ and distance in cm.

For the between objects condition, the luminance of each pixel that belonged to an object (i.e was not part of the background) was manipulated using the following equation:

2.2 $$L_{i}=L_{0}\pm \frac{Z_{i}-Z_{0}}{\beta },$$

where *L*_0_ is the original luminance, *Z_i_* is the mean distance of pixels in object *i*, *Z*_0_ is the viewing distance (50 cm) and *β* determines the degree to which each pixel's luminance is scaled relative to its distance. A value of *β* = 100 cm was used.

#### 2.1.2. Procedure

On each trial, two small circles were presented monocularly on the screen, superimposed on the image. 30 point pairs were chosen for each of three scenes, given a total of 90 pairs. For the within-object conditions, points were chosen randomly, subject to the constraint that they belonged to the same object, and the mean separation between points in the plane of the screen was 2.4 cm, and in three-dimensional space 2.6 cm. For the between-object conditions, points were chosen randomly, subject to the constraint that they belonged to different objects; the mean separation between points in the place of the screen was 7.7 cm, and in three-dimensional space 11.7 cm.

On each trial, a horizontal line was presented on the screen, 6.1 cm below the stimulus, centred horizontally. The length of the line was randomly initialized to a value of between 0 and 4.5 cm. By moving the computer mouse leftwards or rightwards, the observers (*n* = 15) could increase or decrease the length of the line. Their task was to match the length of the line to the distance, in three-dimensional space, between the two points [47]. This task was completed under both monocular and binocular viewing. Under monocular viewing, one image was presented to the participant's right eye and a black screen to the left. This means that there were no binocular cues to the flatness of the viewing screen in the monocular viewing.

The experiment was approved by the University of Essex Research Ethics Committee.

### 2.2. Results

In all cases, settings increased with increasing physical distance, as expected. For the within-object manipulations (figure 5), with monocular depth cues, a regression of set distance against actual distance produced a slope of 1.14 (*p* *<* 0*.*0001) with a reduced correlation, and 1.10 (*p* *<* 0*.*0001) with an enhanced correlation. The fact that these regression slopes are greater than one indicates an overestimation of distance in these two conditions. With binocular depth cues, the slopes were 1.03 (*p* *<* 0*.*0001) for the reduced correlation and 1.02 (*p* *<* 0*.*0001) for the enhanced correlation.

Each setting was divided by the actual distance between those two points in three-dimensional space to give a distance ratio measure. If observers tended to make accurate settings, this ratio would on average be 1. If the manipulation of luminance acted as a depth cue, we would expect larger distance ratios for the enhanced condition, and smaller ratios for the reduced condition. Our results are plotted separately for each of the within-object (figure 5) and between-object (figure 2) conditions. For each condition, data were analysed with a two-way repeated measures ANOVA, with viewing condition (monocular or binocular) and luminance manipulation (reduced or enhanced) as the two factors.

For the within-object condition (figure 5), observers made significantly larger settings under monocular viewing (*F*_1*,*89_ = 19*.*80; *p* *<* 0*.*001). They also made significantly larger settings in the enhanced condition than in the reduced condition (*F*_1*,*89_ = 6*.*93; *p* = 0*.*010). There was no significant interaction (*F*_1*,*89_ = 0*.*498; *p* = 0*.*482). Pairwise *t*-tests showed that the effect of manipulation was significant under monocular viewing (*t*_89_ = 2*.*422; *p* = 0*.*017) but not under binocular viewing (*t*_89_ = 0*.*938; *p* = 0*.*351).

The greater apparent distance in the enhanced condition under monocular viewing reflected a shift in the intercept, rather than an increase in the slope of the function relating actual to perceived distance. To confirm this difference in settings between the two viewing conditions, we performed a regression of set distance in the enhanced and reduced conditions. This showed a significant positive intercept of 0.30 cm (*p* < 0.001) and slope of 0.95 (*p* < 0.001) (figure 5*b*). Under binocular viewing (figure 5*c*), there was also a significant intercept of 0.17 cm (*p* = 0.025) and slope of 0.96 (*p* < 0.001). Individual data are plotted in the supplementary materials. A significant increase in the set length/actual length ratio in the enhanced condition relative to the reduced condition was found for 7 of the 15 participants individually under monocular viewing, compared with 2 out of 15 under binocular viewing.

The lack of a significant interaction means that our data do not provide evidence for a difference in the effect of luminance contrast under monocular and binocular viewing. However, the *t*-tests that we performed also provide evidence for an increase in the average depth setting under monocular but not binocular viewing.

For the between-object condition (figure 2), settings again increased with increasing physical separation. With only monocular depth cues, a regression of set distance against actual distance produced a slope of 0.34 (*p* *<* 0*.*0001) with the reduced correlation, and 0.35 (*p* *<* 0*.*0001) with the enhanced correlation. With binocular depth cues, the slopes were 0.52 (*p* *<* 0*.*0001) with the reduced correlation and 0.53 (*p* *<* 0*.*0001) with the enhanced correlation. These slope values indicate an underestimation of distance in all cases in the between-object condition.

For the between-object condition (figure 2*a*), observers again made significantly smaller settings under binocular viewing (*F*_1*,*89_ = 4.39; *p* = 0*.*039) but there was no effect of luminance manipulation (*F*_1*,*89_ = 0*.*015; *p* = 0*.*904). There was a significant interaction (*F*_1*,*89_ = 4.62; *p* = 0*.*034), reflecting a larger effect of viewing condition with a reduced luminance–distance correlation. Settings were smaller under binocular viewing than monocular viewing with a reduced luminance-distance correlation (*t*_89_ = 2.53; *p* = 0.013), but not with an increased luminance–distance correlation (*t*_89_ = 1.30; *p* = 0.196). In neither the monocular (*t*_89_ = 1.74; *p* = 0.086) nor binocular (*t*_89_ = −1.30; *p* = 0.195) condition was there an effect of image manipulation.

Settings were more accurate (less biased) in the between-object condition than the within-object condition. The two differences between these conditions are firstly that the points are on separate objects rather than the same object in the global condition, and secondly that the distances between the points were larger in this condition.

These results show that the perceived three-dimensional separation between points was larger in the enhanced condition than the reduced condition when image manipulations and judgements were made locally, within objects (figure 5*a*), but not when they were made between objects (figure 2*a*). This is consistent with the use of the dark-is-deep correlation as a cue to depth within local regions of the image.

## 3. Experiment two

Observers were presented with depth variations defined by luminance and binocular disparity. Depth from disparity was provided by using random dot stereograms, and depth from luminance by varying the luminance of the dots. This ensured that both cues provided reliable depth information. Stimuli were presented either with a consistent (darker regions farther away) or inconsistent (darker regions closer) depth sign. Contrast and disparity were independently manipulated. These stimuli are similar to those used in a previous experiment to assess how these cues are used to estimate the spatial location of the depth peak of a surface [30]. In the current study, the task was to set the length of a horizontal line on the screen to match the perceived distance in depth between the nearest and farthest points in the scene.

The mandatory-combination model predicts that depth will increase with disparity, increase with contrast in the consistent condition, and decrease with contrast in the inconsistent condition. The consistency-dependent combination models predicts that depth will increase with disparity in both conditions, and increase with contrast only in the consistent condition.

### 3.1. Methods

#### 3.1.1. Materials

**Figure 6.**
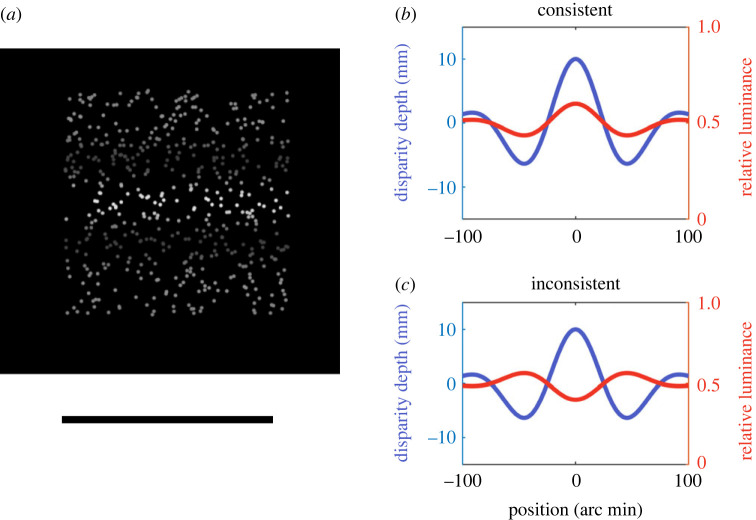
(*a*) The second experiment used random dot stereograms in which the luminance of the dots, as well as their binocular disparity, was varied. Luminance and disparity were either (*b*) consistent, so that nearer points were brighter or (*c*) inconsistent. The observers' task was to set the length of the horizontal line to match the apparent distance in depth between the nearest and furthest point on the surface.

Stimuli were random dot stereograms (figure 6*a*). The individual elements were anti-aliased circular dots with a diameter of 2 arc min. 500 dots were presented in a 250 arc min central square window. The luminance of the dots was varied according to a horizontal Gabor profile, with a spatial frequency of 0.6 cycles/degree and a standard deviation of 50 arc min. The binocular disparity of the dots was also varied according to the same Gabor profile, with an amplitude of 0, 2.7, 7.4, 11.2, 14.9 or 18.6 arc min. In separate blocks of trials, this created either ‘bump’ stimuli in which the central point was closest to the observer, or ‘dip’ stimuli, in which it was furthest away, depending on the phase of the disparity modulation (figure 6*b*).

The mean luminance of the dots was 50 cdm^−2^ and their luminance was varied around this level with a Michelson contrast of 0, 0.1 or 0.2. For the non-zero contrast modulations, the central region was brighter than average on half the trials and darker on the other half. This created stimuli in which the luminance and disparity information, when considered as depth cues, were either consistent (when nearer regions were brighter) or inconsistent (when nearer regions were darker). Dots were presented against a black background.

#### 3.1.2. Procedure

Observers (*n* = 19) performed a similar distance judgement task as in the first experiment. In this experiment, their task was to match the length of the line to the perceived depth of the stimulus (the distance in depth between the closest and furthest point). Bump and dip stimuli were presented in separate blocks of trials. In each block, all 30 combinations of the 6 disparities and 5 contrast modulations were presented once each, in random order. Each observer completed 5 repetitions of each block.

### 3.2. Results

**Figure 7.**
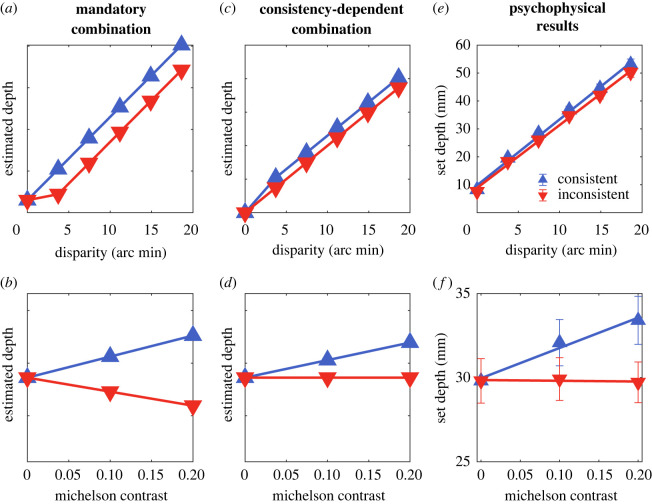
Predicted and measured depth in the second experiment as a function of disparity (top row) and contrast (bottom row). Data are plotted separately for cases in which the sign of depth is consistent or inconsistent. The lefthand column shows predictions for the mandatory-combination model (equation (1.1)). Since the exact predictions will depend on the weights used, they are plotted in arbitrary depth units. In all cases, data show the total effect of each cue (disparity or contrast) averaged across all other values of the other cue, which had a mean value of zero across all stimuli (*a*) Depth is predicted to increase with disparity, and to be greater when cues are consistent (and add) than when they are inconsistent (and subtract). The deviation from linearity shown for inconsistent cues for small values reflects the change in sign of depth (relative to that predicted by disparity) and the calculation of absolute depth (which is what the participants reported). (*b*) In this model, depth is predicted to increase with increasing contrast when the cues are consistent but to decrease with increasing contrast when the cues are inconsistent. The middle column shows the predictions for the consistency-dependent combination model (equation (1.2)). (*c*) Estimated depth is again predicted to increase with disparity, and to be greater for consistent cues. (*d*) When the cues are consistent, depth is predicted to increase with increasing contrast. When they are inconsistent, depth is not affected by changes in contrast. (*e*) Psychophysical results show that depth increases with disparity for both consistent and inconsistent stimuli. (*f*) For consistent stimuli, perceived depth increases with contrast. For inconsistent stimuli, perceived depth is unaffected by contrast. These results are predicted by the consistency-dependent combination model, but not the mandatory-combination model. Symbols show the mean results across participants and conditions, and error bars show ±1 standard deviation.

Perceived depth is plotted as a function of disparity in figure 7*e*. Depth settings are averaged over observers, contrasts and repetitions. Perceived depth increased with increasing disparity in both the consistent and inconsistent conditions. Perceived depth is plotted as a function of luminance contrast in figure 7*f*. Depth settings are averaged over observers, disparities and repetitions. When the two cues were consistent, perceived depth increased with increasing contrast, as predicted. However, when disparity and contrast were inconsistent, there was no effect of contrast on depth settings, suggesting that variations in perceived depth in this case were determined purely by the unambiguous cue of binocular disparity. These results are predicted by the consistency-dependent combination model, but not the mandatory-combination model.

These models were tested using a mixed effects regression to predict apparent depth from disparity and contrast, as fixed covariates, and shape (bump or dip) as a categorical factor. The latter was included in case the sign of disparity affected the magnitude of perceived depth. The random effect structure was determined by testing all combinations of a random intercept, and random slopes (across disparity, contrast and shape), and selecting the model that provided the smallest AIC. The chosen model, which included random intercepts and slopes for disparity and contrast, was then fit with a restricted maximum-likelihood procedure, and the fixed-effects denominator degrees of freedom were calculated using a Satterthwaite approximation [48]. Models were fit separately for the consistent and inconsistent stimuli. The mandatory combination model predicts that the disparity parameter will be positive and that the contrast parameter will be positive for consistent stimuli, and negative for inconsistent stimuli. The consistency-dependent combination model predicts that the disparity parameter will be positive and that the contrast parameter will be positive for consistent stimuli, and zero for inconsistent stimuli.

For the consistent-cues stimuli, depth increased with increasing disparity (*β* = 32*.*41; *t*_19_ = 9*.*38; *p* *<* 0*.*001) and luminance contrast (*β* = 17*.*99; *t*_19_ = 3*.*41; *p* = 0*.*0029) and did not differ between bumps and dips (*β* = 0*.*50; *t*_627_ = 0*.*92; *p* = 0*.*358). For the inconsistent-cues stimuli, depth increased with increasing disparity (*β* = 30*.*92; *t*_19_ = 9*.*72; *p* *<* 0*.*001), but not luminance contrast (*β* = −0*.*43; *t*_154*.*27_ = −0*.*133; *p* = 0*.*89) or shape (*β* = 61; *t*_646_ = 1.18; *p* *=* 0*.*237). Thus, when luminance contrast conflicted with the unambiguous cue of binocular disparity, it did not contribute to variations in apparent depth. The difference between our results and an earlier study [3] which found that contrast dominated binocular disparity is most likely to reflect the less reliable disparity cue in their stimuli. Our results show that the negative correlation between luminance and distance, as found under diffuse lighting, provides a depth cue that is combined with depth from binocular disparity, in a way that is consistent with the simultaneous estimation of surface depth and reflectance variations.

We also analysed the data for individual participants. This allowed us to establish whether anyone showed significant effects that ran contrary to those found across participants. All but one showed a significant effect of disparity, with perceived depth increasing with disparity magnitude for both the consistent and inconsistent conditions. For luminance contrast, 6 of the 19 participants showed a significant increase in perceived depth with increasing contrast in the consistent condition, and none showed a significant decrease. In the inconsistent condition, no participant showed a significant effect of contrast in either direction. These results illustrate the benefits of the pooling of data across participants prior to the regression analysis [49].

### 3.3. Discussion

We have shown that the negative correlation between luminance and disparity that results from diffuse lighting influences the perception of depth. This effect is evident not just in simple corrugations, but in complex scenes containing rich monocular depth cues. For these scenes, created from three-dimensional scans of real objects, there was a strong correlation between observers' judgements and the actual three-dimensional distance between target points in all conditions. When the two points were on the same object, observers tended to make smaller depth settings in the binocular viewing condition. Since settings were too large in the monocular case, this represents more accurate perception of depth when binocular cues are available. Manipulation of the luminance of pixels within objects influenced perceived three-dimensional structure, but only when scenes were viewed monocularly. When the two points were on different objects, settings were lower, and again smaller under binocular viewing, and there was no effect of the luminance manipulation. This lack of an effect of more global manipulations of luminance, between objects, is consistent with the role of luminance variations as a local depth cue. The difference in the accuracy of depth between the within- and between-object cases is beyond the scope of the current investigation. However, one potential explanation is that, in the between-object condition points were unlikely to be separated by a monotonic variation in depth, which is known to influence depth judgements [50–52].

In experiment 1, we used scenes containing multiple objects against a mid-grey background. We also chose to include multiple sources of lighting, and to artificially manipulate the correlation between luminance and distance through directly altering the luminance of individual pixels. This design was intended to build on the similar manipulation used in a previous study [21] rather than to assess the source of the negative luminance-depth correlation directly. It is possible therefore that stronger influences of this manipulation may be found in other situations. For example, a scene in which the observer was looking into a bush or dense forest would be expected to create a global luminance-depth correlation, in which the furthest points were also the darkest. This contrasts with our neutral use of a mid-grey luminance for a background with unspecified surface structure. Scenes that contained direct evidence that the luminance-depth correlation is the result of diffuse lighting and ambient occlusion might also be expected to influence depth more strongly. If so, this would demonstrate that observers were capable of making a sophisticated interpretation of this contextual information. By contrast, our results and those of Cooper & Norcia [20] also demonstrate the more direct influence of the luminance-depth correlation on the perception of depth in the absence of these context effects.

Enhancing the local luminance cue to depth increases observers’ sense that they are looking at a three-dimensional scene [21], but that study did not measure the amount of depth perceived. This qualitative three-dimensional judgement may be influenced by multiple factors [53] and a clear distinction has been drawn between the qualitative impression and quantitative magnitude of perceived depth [22,28,54,55]. This distinction has been addressed using stimuli that were the sum of a disparity-defined sinusoidal luminance profile, and a sinusoidal luminance profile that had either the same or double the frequency [24]. In addition, the phase of the luminance profile was either consistent or inconsistent with disparity-defined depth, when considered as a shape-from-shading cue. They asked observers to judge the three-dimensional realism of the surface or its apparent depth. While participants chose the consistent stimuli as the more realistic, the stimulus that was perceived as the deeper differed between the two spatial frequencies. These results demonstrate that judgements of the strength of the three-dimensional experience, and the amount of perceived depth, can be dissociated. The results were interpreted as reflecting the role of the luminance cues in segmenting the image [24]. This interpretation emphasizes the complexity and ambiguity of how cues contribute to the overall interpretation of the image.

Depth discrimination thresholds also improve when luminance is consistent with a dark-is-deep correlation [20], demonstrating that this depth-luminance correlation contributes to categorical, near/far judgements in cluttered scenes containing multiple identical objects. We have shown that local luminance variation affects the quantitative magnitude of perceived depth, thus establishing it as a cue to depth magnitude in complex scenes, not just depth discrimination. Critically, in experiment 2 we also showed that increasing luminance contrast led to an increase in perceived depth when the shading cue was consistent with depth from binocular disparity but had no effect when the two cues were inconsistent. By contrast to this the perceived spatial localization of a depth profile has been found to appear consistent with the fusion of the two cues into a single depth perception [30], even when they were inconsistent. This meant that a threshold for spatial localization could not be measured when the cues signalled equal and opposite depths and thus cancelled. This study used a higher luminance contrast (50%) than the 20% used here, which may have contributed to this difference. However, even at the lower luminance contrast used in the current study, a clear asymmetry is evident in the result for consistent and inconsistent depth cues.

In a study in which shape was defined by both binocular disparity and shading, and the two cues were put in conflict, depth perception was consistent with a standard, mandatory cue combination model [19]. In this study, both directional and ambient lighting were present, and a photorealistic rendering process was used. The cue perturbation method used, in which the shapes defined by disparity and shading were independently varied, differs from the approach adopted in our first experiment, in which we specifically manipulated the correlation between luminance and depth. Our approach is intended to isolate the influence of this image property while keeping all other aspects of the stimuli unchanged [56].

In addition to the effects of luminance variations on perceived depth, it is also known that the overall contrast of a stimulus affects the amount of depth from disparity [17]. In that study, for a given disparity, perceived depth in random dot stereograms increased with increasing contrast, for stimuli in which overall contrast was varied, and the luminance of individual pixels was not structured to correlate with disparity or to provide a depth cue in its own right. The depth-dependent manipulation of luminance in our stimuli did have a small effect on RMS contrast, which could have influenced apparent depth. However, if the effect of luminance on perceived depth was driven purely by overall contrast, we would have expected the same, positive effect in both the consistent and inconsistent conditions, whereas we only found an effect in the consistent-cues condition. The role of luminance contrast as a depth cue thus provides a coherent explanation of our results, whereas an interpretation in terms of the effect of contrast does not.

The combination of the depth cues of binocular disparity and texture occurs in higher visual areas, such as hMT+, rather than in early cortical areas such as V1 and V2 [57]. From this, we might predict a relatively late stage of combination of luminance-based cues with other depth cues. However, the negative correlation between luminance and depth is reflected in the tuning of cells in V1 to these two dimensions [58]. This tuning is consistent with different distributions of expected binocular disparities in natural images for locally bright and locally dark points [21]. As such, the presence of this correlation in early visual processing would reflect an efficient encoding of visual information, rather than directly contributing to the combination of the two as depth cues. This latter process is complicated by the fact that the two need to be scaled in different ways, to take account of the viewing geometry in the case of binocular disparity, and assumptions about the lighting conditions in the case of luminance. This again is consistent with this combination occurring at higher cortical levels of visual processing.

The consistency-dependent combination of disparity and luminance in the estimation of depth could be implemented via an explicit decision stage, as in causal inference models [35]. However, an added complexity in the case of luminance contrast is that the ambiguity relates to whether variations in luminance result from changes in depth or reflectance. This can be incorporated into Bayesian models using two-dimensional prior and likelihood functions, defined over both depth and reflectance. Our consistency-dependent combination model assumes that luminance variations relate to changes in depth or reflectance, but not both. This assumption would constrain the form of the Bayesian prior. It is also possible however that reflectance and depth may covary. An example of this is countershading, in which the estimation of shape-from-shading is complicated by a natural variation between surface orientation and reflectance in the colouring of some prey animals [59]. Such considerations illustrate the difficulty inherent in the interpretation of shading cues to depth. Despite this complexity, our results show that the ‘dark-is-deep’ correlation contributes to the perception of metric depth and that this contribution is combined with depth estimated from binocular disparity. The results highlight the importance of understanding the complexities of lighting beyond the consideration of the direct reflection of light from a single direction, for the perception of depth as well as the subjective realism of three-dimensional scenes. Adopting more complex lighting models will thus contribute to the accuracy as well as the aesthetic appearance of three-dimensional graphics.

## Data Availability

Data are available via this link: https://figshare.com/s/c2671e97ec8e60629fda. The data are provided in electronic supplementary material [60].
